# First Synthesis of Ergotamine-^13^CD_3_ and Ergotaminine-^13^CD_3_ from Unlabeled Ergotamine

**DOI:** 10.3390/toxins16040199

**Published:** 2024-04-20

**Authors:** Sven-Oliver Herter, Hajo Haase, Matthias Koch

**Affiliations:** 1 Division 1.7, Organic Trace and Food Analysis, Bundesanstalt für Materialforschung und-prüfung (BAM), Richard-Willstätter-Str. 11, 12489 Berlin, Germany; sven-oliver.herter@bam.de; 2 Department of Food Chemistry and Toxicology, Technische Universität Berlin, Gustav-Meyer-Allee 25, 13355 Berlin, Germany; haase@tu-berlin.de

**Keywords:** priority ergot alkaloids, mycotoxins, isotopically labeled internal standard, maximum levels, standardized method

## Abstract

Ergot alkaloids (EAs) formed by *Claviceps* fungi are one of the most common food contaminants worldwide, affecting cereals such as rye, wheat, and barley. To accurately determine the level of contamination and to monitor EAs maximum levels set by the European Union, the six most common EAs (so-called priority EAs) and their corresponding epimers are quantified using high-performance liquid chromatography coupled with tandem mass spectrometry (HPLC-MS/MS). The quantification of EAs in complex food matrices without appropriate internal standards is challenging but currently carried out in the standard method EN 17425:2021 due to their commercial unavailability. To address the need for isotopically labeled EAs, we focus on two semi-synthetic approaches for the synthesis of these reference standards. Therefore, we investigate the feasibility of the *N*^6^-demethylation of native ergotamine to yield norergotamine, which can subsequently be remethylated with an isotopically labeled methylating reagent, such as iodomethane (^13^CD_3_-I), to yield isotopically labeled ergotamine and its *C*^8^-epimer ergotaminine. Testing the isotopically labeled ergotamine/-inine against native ergotamine/-inine with HPLC coupled to high-resolution HR-MS/MS proved the structure of ergotamine-^13^CD_3_ and ergotaminine-^13^CD_3_. Thus, for the first time, we can describe their synthesis from unlabeled, native ergotamine. Furthermore, this approach is promising as a universal way to synthesize other isotopically labeled EAs.

## 1. Introduction

Providing safe food for an ever-growing world population is one of the great challenges of the 21st century. At 2.8 billion tons per year, cereals are the world’s most important staple food. However, climate change and cereal contamination can have serious economic and health consequences. The most common cereal contaminants are mycotoxins. Depending on the level of contamination, they can cause serious short- and long-term health problems. Recent studies have examined mycotoxin contamination in food and feed around the world. The results show that in 60–80% of all tested cereals, at least one mycotoxin was detected [1,2,3]. 

**Figure 1:**
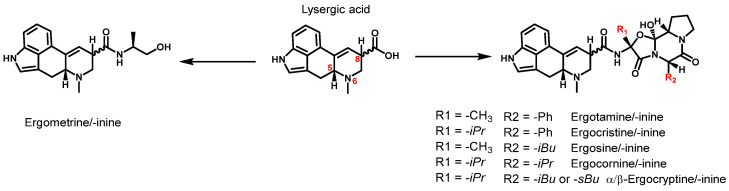
Structure of the 6 most abundant ergot alkaloids and their *C*^8^-stereoisomer based on the structure of lysergic acid (R: -Ph = phenyl group; -iPr = isopropyl group; -iBu = isobutyl group; -sBu = secbutyl group). The structure of lysergic acid shows the locants for the *C*^5^-, *N*^6^-, and *C*^8^-atoms.

A toxicologically relevant group of mycotoxins are ergot alkaloids (EAs). These are secondary metabolites produced by several ubiquitous fungi of the genus *Claviceps*. They grow preferentially on rye and wheat but can also infect other cereals such as triticale, barley, and millet [4]. *Claviceps purpurea* is the most common ergot fungus in Europe and forms a hardened mycelium called sclerotia in preparation for winter [5,6]. Sclerotia accumulate biomolecules such as alkaloids and lipids for the fruiting phase of the fungus in spring [7]. Toxic EAs are introduced into food and feed through the harvest of sclerotium-infested grain and subsequent processing, e.g., in mills. Figure 1 shows the six most commonly monitored EAs and their corresponding *C*^8^-stereoisomers.

The EAs presented are all lysergic acid amides and can be divided into two distinct groups: simple lysergic acid derivatives, such as ergometrine, and the group of ergopeptines with a tricyclic peptide ring. The corresponding stereoisomers are formed by isomerization at the *C*^8^-atom of the lysergic acid moiety. Therefore, the *C*^8^-(*R*) configuration is referred to as ergopeptines (e.g., ergotamine) and the *C*^8^-(*S*) as ergopeptinines (e.g., ergotaminine). Both the *R*- and *S*-form exhibit different biological activities, with ergopeptines being more toxic than ergopeptinines [8,9]. However, since both epimers are interconvertible, it is crucial to quantify both epimers in cereal-based products to determine the level of contamination and toxicity.

The European Union has first established maximum levels for certain EAs in foodstuffs due to their toxicological relevance, as outlined in Commission Regulation (EC) No 2021/1399 amending Regulation No 1881/2006. The maximum level of EAs in foodstuffs refers to the lower-bound sum of the six priority EAs and their epimers depicted in Figure 1. The current maximum level ranges from 500 µg/kg in rye milling products down to 20 µg/kg in processed cereal-based food for infants and young children [10].

Simultaneously, EN 17425 (2021) was published as the first standardized method for quantifying priority EAs using high-performance liquid chromatography with tandem mass spectrometry (HPLC-MS/MS) [11]. While this method is a step in the right direction, it still has inherent problems. In HPLC-MS/MS, the matrix co-eluting with the analyte molecules can directly affect their ionization, resulting in an increase or decrease in the analyte signal (known as the matrix effect) [12,13]. This effect directly impacts the accuracy of the measurement, potentially leading to an over- or underestimation of EA contamination [14,15]. To overcome this issue, an internal standard (ISTD) can be used. In HPLC-MS/MS, a suitable ISTD is a chemically similar compound to the analyte with comparable retention time, ionization response, and fragmentation pattern [16]. Taking these criteria into account, isotopically labeled analytes (^2^H, ^13^C, and ^15^N) are ideal ISTDs for MS-based analytical methods. Therefore, isotopically labeled ISTDs are preferably used for food, environmental, and bioanalytical methods to improve quantification. Although there is a need for isotopically labeled ISTDs, their use depends on availability. In the case of the six priority EAs and their epimers, isotopically labeled ISTDs are not fully commercially available, which limits the current standardized method [17]. This urgent need will be further aggravated by the reduction in limit values for EAs in various cereal products in 2024 [18].

Various strategies can be employed to address the issue of unavailable isotopically labeled EAs. Currently, only the simple lysergic acid derivatives ergometrine and ergometrinine exist as ^13^CD_3_-ISTDs, but not the ergopeptines [17,19]. To overcome this problem, one possible strategy involves synthesizing the cyclic peptide ring and then coupling it to an isotopically labeled lysergic acid moiety. This procedure has already been described in the literature for unlabeled EAs [20,21]. However, the process of total synthesis is time-consuming and complex, resulting in low overall yields and high production costs. Another approach begins with an unlabeled native EA, followed by subsequent chemical modifications to obtain an isotopically labeled EA. It is important to consider the specific position to be modified and select a group that is large enough to accommodate a mass shift (∆m) of 4 Da between the native and labeled EAs. With a mass difference of 4 Da compared to 3 Da or less, the isotopic distribution of the native EA has less influence on the signal of the isotopically labeled EA. As this directly affects quantification by stable isotope dilution analysis, the aim is to maximize the mass difference between native and isotopically labeled EAs. Increasing the mass shift beyond 4 Da would further decrease the influence on the signal of the isotopically labeled EA but would also complicate the choice of a specific position to be modified. Because all priority EAs share the lysergic acid structure methylated at the *N*^6^-position, this study focuses on the feasibility of the N-demethylation of native ergotamine at the *N*^6^-position [22]. As a major representative of the ergopeptine group, we have chosen ergotamine for this feasibility study. Subsequent remethylation at the *N*^6^-atom with an isotopically labeled methylation reagent should yield ergotamine/-inine-^13^CD_3_.

## 2. Results

### 2.1. Electrochemical N-Demethylation

Electrochemistry is widely considered to be a mild, green, and atom-efficient tool for synthesis, especially for oxidation and reduction reactions where no additional reagent is required [23]. For example, electrochemically anodic oxidative N-demethylation has been studied for the synthesis of noropiates and nortropanes, which are important intermediates in drug synthesis [22,24]. Therefore, we decided to use electrochemistry to determine the feasibility of the *N*^6^-demethylation of ergotamine. Various working and auxiliary electrodes, electrolytes, and solvents were tested for N-demethylation (Table 1). The reaction solutions were pumped through the electrochemical cell, and the voltage was ramped up continuously from 0 to 1.2 V. The reaction products were analyzed directly using mass spectrometry and via HPLC-MS.

**Figure 2:**
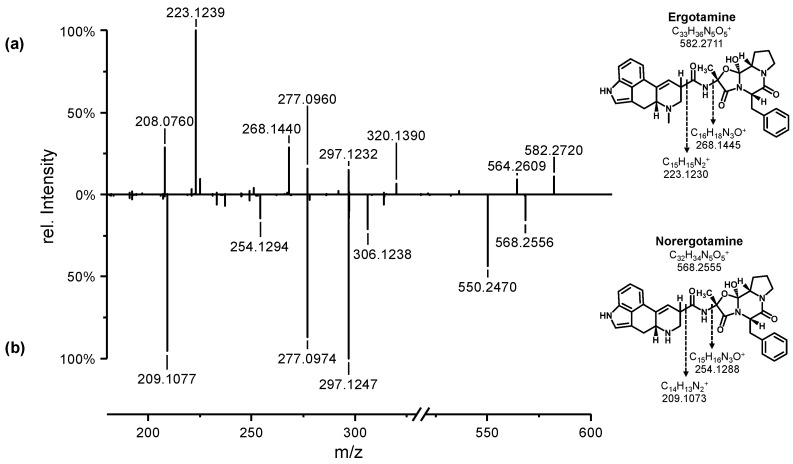
Positive electrospray ionization high-resolution tandem mass spectra [M + H]^+^ of (**a**) ergotamine and (**b**) norergotamine. The structures are displayed on the right-hand side, along with the theoretical exact masses for the molecular ion [M + H]^+^ and major fragment ions containing the *N*^6^-atom.

When the reaction was carried out in methanol, only a methoxy adduct of ergotamine was detected under all tested conditions. As a result, we decided to test acetonitrile instead of methanol. No methoxy adduct was detected when acetonitrile was used. However, significant side reactions, such as hydroxylation and dehydrogenation, were observed. Using ammonium acetate or ammonium carbonate as an electrolyte with a glassy carbon (GC) electrode at a voltage of 0.6 V, we were able to detect a product with an appropriate nominal mass-to-charge ratio (*m*/*z*) of 568. This *m*/*z* corresponds to the theoretical nominal mass of *N*^6^-demethylated ergotamine (norergotamine). The use of formic acid as an electrolyte did not result in the formation of a product with *m*/*z* 568. A sample of the reaction solution was collected and analyzed using high-resolution tandem mass spectrometry (HRMS). Figure 2 displays the tandem mass spectra of unreacted ergotamine and the product with *m*/*z* 568.

The high-resolution tandem mass spectrum of ergotamine in Figure 2a is consistent with previous reports in the literature [25,26]. Figure 2 compares the fragmentation of ergotamine and norergotamine. Both spectra exhibit a similar fragmentation pattern; however, the different intensities of certain fragment ions can be attributed to the lower energy required for the fragmentation of norergotamine. Fragment ions with ∆*m*/*z* values of 14.0156 and 14.0146 were detected for norergotamine (209.1077; 254.1294) and ergotamine (223.1239; 268.1440). These fragments correspond to the fragmentation of the alky-carbonyl or amino-alkyl bond of the amide between the lysergic acid and the tricyclic peptide ring. For these fragment ions, the observed difference in the *m*/*z* values between ergotamine and norergotamine suggests the demethylation of the *N*^6^-atom (-CH_2_; theoretical *m*/*z* 14.0156). The high-resolution masses of the precursor and product ions agreed with the theoretical values, giving us confidence that the electrochemical *N*^6^-demethylation of ergotamine had been achieved on an analytical scale.

### 2.2. Synthesis of Norergotamine/-inine via an Iron-Catalyzed N-Demethylation Reaction

**Figure 3:**
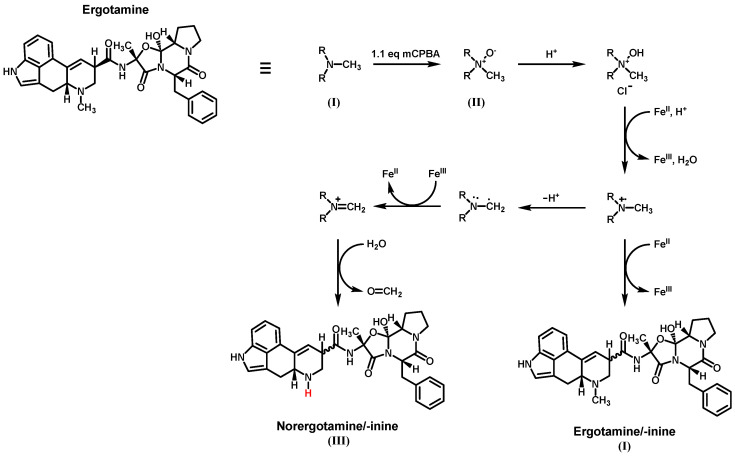
Reaction mechanism for the *N*^6^-demethylation of ergotamine using an iron-catalyzed N-demethylation reaction.

The N-demethylation of amines is a common reaction type in the metabolic pathway of xenobiotics and is also used in the synthesis of pharmaceuticals, agrochemicals, and fine chemicals. Frequently used reactions include the *von Braun* reaction or the use of chloroformates, but both reactions utilize highly reactive or toxic chemicals and harsh reaction conditions [27,28]. Therefore, we investigated the feasibility of N-demethylation via an iron-catalyzed reaction that does not involve highly reactive or toxic chemicals and does not require high temperatures. Iron was chosen as the catalyst due to its demonstrated catalytic activity for the N-demethylation reaction, broad availability, low cost, and environmental friendliness compared to other approaches that utilize transition metals such as palladium or platinum [29]. For our first demethylation attempt, we chose ergotamine as the primary example of ergopeptines. Figure 3 illustrates the reaction mechanism using ergotamine as an example. The first step involves the conversion of the tertiary amine (I) into the corresponding N-oxide (II) using an organic or inorganic peroxide. Hydrogen peroxide (H_2_O_2_), *meta*-chloroperoxybenzoic acid (mCPBA), and potassium peroxymonosulfate (oxone^®^, KHSO_5_) were tested for this purpose. However, H_2_O_2_ and KHSO_5_ were found to be unsuitable due to incomplete conversion of ergotamine or significant by-product formation. The use of mCPBA resulted in the main oxidation product (II), with only minor by-product formation.

The N-oxide can either be isolated or used directly in the second step. Therefore, an acid (such as sulfuric acid or hydrochloric acid) and an Fe^II^ salt or Fe^0^ (iron powder) are added to the reaction mixture. Fe^0^ is oxidized and forms the active Fe^II^ species in situ. A redox pair of Fe^II^/Fe^III^, involving two consecutive one-electron transfers, is believed to be responsible for the sequential reduction of the N-oxide [29]. To test this for ergotamine, we experimented with iron(II) sulfate, iron(II) chloride, ferrocene, and iron powder in various solvents and concentrations for the reduction of ergotamine-N-oxide. Over a reaction time of 24-h, the tested iron salts resulted in only minor product formation, while ferrocene primarily promoted the formation of ergotamine/-inine (I) as a major by-product and only showed minor formation of norergotamine (III). When using iron powder in aprotic solvents (tetrahydrofuran and dichloromethane), we mainly obtained the parent tertiary amine (I). However, changing to a protic solvent like methanol resulted in substantial formation of the N-demethylated product. During the formation of the desired product, significant epimerization was observed for the N-demethylated product (III) and the main by-product/educt (I). This epimerization is widely described in the literature and is believed to be caused by a combination of low or high pH, heat, and the use of a protic solvent during the reaction [30]. Preparative LC was used to isolate both epimers, norergotamine and norergotaminine, in milligrams for further reactions.

### 2.3. Synthesis of Ergotamine-^13^CD_3_ and Ergotaminine-^13^CD_3_

**Figure 4:**
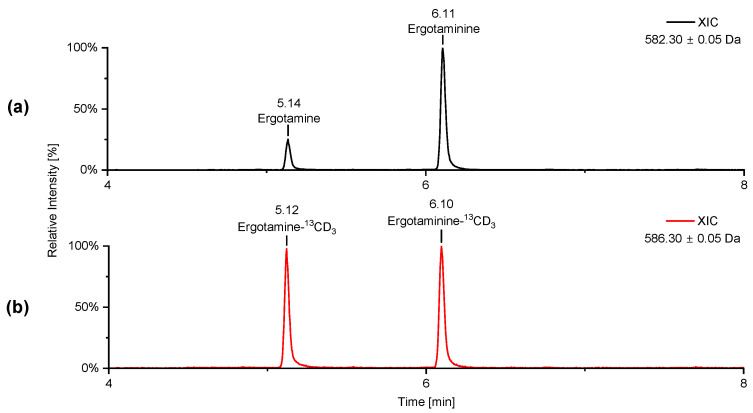
Extracted-ion chromatogram (XIC) [M + H]^+^ of (**a**) unlabeled ergotamine and ergotaminine and (**b**) isotopically labeled ergotamine-^13^CD_3_ and ergotaminine-^13^CD_3._

**Figure 5:**
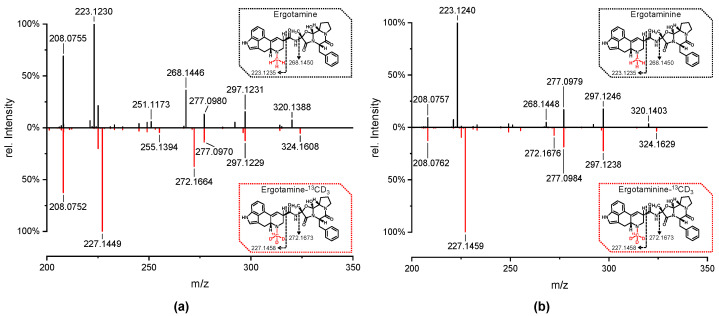
Positive electrospray ionization high-resolution tandem mass spectra [M + H]^+^ of (**a**) unlabeled ergotamine (black) and isotopically labeled ergotamine-^13^CD_3_ (red) and (**b**) unlabeled ergotaminine (black) and isotopically labeled ergotaminine-^13^CD_3_ (red). The structures show the *R*/*S*-configuration at the *C*^8^-position, along with the theoretical exact masses of the major fragment ions containing the *N*^6^-atom.

Iodomethane-^13^CD_3_ was used to methylate the crude products of norergotamine and norergotaminine. The resulting isotopically labeled ergotamine and ergotaminine were purified using preparative HPLC to remove any unreacted norergotamine/-inine. This process resulted in epimerically pure solutions of the isotopically labeled ergotamine and ergotaminine. To verify this, a sample of unlabeled ergotamine/-inine in methanol was spiked with isotopically labeled ergotamine/-inine and analyzed using HPLC-HR-MS/MS (Figure 4 and Figure 5).

Figure 4 displays the extracted-ion chromatogram (XIC) of both labeled and unlabeled ergotamine and ergotaminine. The peaks in the chromatogram align perfectly for both labeled and unlabeled ergotamine/-inine. The HR-MS/MS spectra for both epimers are identical for the labeled and unlabeled substances. Notably, a distinct mass shift of ∆m = 4 Da is observed in the isotopically labeled molecules, especially within the fragment ions containing the labeled *N*^6^-atom.

## 3. Discussion

We tested the feasibility of the *N*^6^-demethylation of ergotamine using electrochemical methods inspired by the literature. A broad spectrum of research has been published in this field for different substances, ranging from metabolomic studies at the microgram scale to multi-gram approaches [31,32,33]. Our experiments have demonstrated that EAs undergo N-demethylation under electrochemical oxidative conditions. However, we encountered significant challenges when attempting to scale up from a µg to a mg scale. The problem of by-product formation has not been overcome. Approximately 85% of the products formed by ergotamine are oxidation products, primarily hydroxylation and the formation of double bonds at various positions. These results led us to investigate alternative methods for achieving *N*^6^-demethylation.

The reaction of ergotamine with mCPBA yielded mostly the desired *N*^6^-oxidation product. The highest conversion rate for demethylation was achieved using iron powder in 10-fold excess. Unreacted iron powder was easily removed by centrifugation, and the secondary amine was purified using preparative HPLC. The HR-MS/MS experiments of the formed demethylated product show a mass defect of 14 Da for specific fragment ions containing the *N*^6^-atom, which matches the HR-MS/MS spectra of the electrochemically synthesized product.

The subsequent reaction of norergotamine and norergotaminine with ^13^CD_3_-I led to the formation of both isotopically labeled epimers of ergotamine. The data presented in Figure 4 and Figure 5 achieve the highest level of confirmation according to the nomenclature of Schymanski et al., confirming the structure of isotopically labeled ergotamine and ergotaminine [34]. Thus, for the first time, we were able to describe the synthesis of isotopically labeled ergotamine and its epimer from unlabeled ergotamine.

To ensure confident monitoring of all priority EAs and their epimers in a standardized method, it is necessary to have isotopically labeled standards for all six of them and their corresponding epimers. Therefore, the approach used targets a shared structural feature among all priority EAs. Consequently, we are confident that we have developed a universal strategy to address the unavailability of these isotopically labeled EAs.

## 4. Materials and Methods

### 4.1. Chemicals and Equipment

All chemicals were used without further purification. Ergotamine-D-tartrate, 3-chloroperoxybenzoic acid (mCPBA, ≤77%), iron powder (for analysis, 10 µm), *N*,*N*-diisopropylethylamine (≥99%), and ferrocene (for synthesis) were purchased from Sigma-Aldrich (Merck KGaA, Darmstadt, Germany). Iron(II) sulfate heptahydrate, iron(II) chloride tetrahydrate, and *tri*-sodium phosphate-12-hydrate were bought from Riedel-de-Haën (Honeywell, Charlotte, NC, USA). Ammonium formate (NH_4_OOCH, Honeywell, Charlotte, NC, USA), ammonium carbonate ((NH_4_)_2_CO_3_, Thermo Fisher Scientific, Waltham, MA, USA), and formic acid (FA, Carlo Erba, Emmendingen, Germany) were bought as electrolytes. Methanol (MeOH), iso-propanol (iPrOH), dichloromethane (DCM), acetone (ACE), tetrahydrofurane (THF), and acetonitrile (ACN) were all LC-grade or higher and obtained from Th. Geyer (Renningen, Germany). Isotopically labeled iodomethane (^13^CD_3_-I) was bought from Eurisotop (Saint-Aubin, France).

The electrochemical experiments were performed in a three-electrode cell (μ-prepcell™, Antec, Zoeterwoude, The Netherlands) connected to a ROXY™ potentiostat controlled using Dialogue software (Version 2.02.199). The µ-prepcell consists of a working electrode (boron-doped diamond (BDE), glassy carbon (GC), or platinum (PT)), a reference electrode (Pd/H2, HyREF™), and an auxiliary electrode (conductive polymeric inlet block). The reaction solution was pumped through the flow cell at a flow rate of 20 µL/min with a syringe pump (KD Scientific Inc., Holliston, MA, USA). The electrochemical cell was connected to a 6130 quadrupole MS (Agilent, Waldbronn, Germany).

Measurements for reaction control were carried out on an Agilent 1290 Infinity HPLC system (Agilent, Waldbronn, Germany) coupled to a 6130 quadrupole MS (Agilent, Waldbronn, Germany). For the separation of reaction products, a Phenomenex Gemini-NX C18 (150 × 2.0 mm; 3 µm) column was used.

Preparative LC was performed on an Agilent (Waldbronn, Germany) LC system consisting of a 1260 Infinity quaternary pump, a 1200 Autosampler, and a column oven coupled to an Agilent 1200 Diode Array Detector with the wavelength set to 254 nm. The Foxy R1 Fraction Collector (Teledyne Isco, Lincoln, NE, USA) was used to automate sample fractionation. A Knauer Eurospher II 100-5 C18 P (250 × 4 mm; 5 µm) column was used.

Volatile solvents were removed by nitrogen blowdown in an Reacti-Therm™ Heating and Stirring Module (Thermo Fischer Scientific, Waltham, MA, USA). Purified products were dried in a rotary vacuum concentrator RVC 2-25 CDplus (Christ, Osterode am Harz, Germany). For thermoshaking, an HLC MHR-13 thermoshaker (Hettich, Tübingen, Germany) was used.

### 4.2. HPLC-HR-MS/MS

High-resolution mass spectra were measured on a TripleTOF 6600 mass spectrometer (Sciex, Darmstadt, Germany) coupled to a 1290 Infinity II system (Agilent, Waldbronn, Germany). For the measurements, a Phenomenex Kinetex Evo C18 (100 × 2.1 mm; 2.6 µm) column was used. Table 2 presents the TripleTOF parameters and Table 3 the HPLC parameters that were used. MS/MS experiments were conducted using information-dependent acquisition (IDA). The MS/MS spectra were recalibrated based on the theoretical *m*/*z* of the precursor ion. Formulae for the measured product-ion masses were calculated using a threshold of ±5 ppm deviation (Supplementary Material, Table S1). Supplementary Material Figure S1 shows the potential structures of ergotamine/-inine-^13^CD_3_ product ions produced by MS/MS. Supplementary Material Figures S2 and S3 show the full HR-MS/MS spectra of unlabeled and isotopically labeled ergotamine and ergotaminine. Supplementary Material Figure S4 shows the full extracted-ion chromatogram for labeled and isotopically labeled ergotamine and ergotaminine.

### 4.3. Electrochemical Experiments

Ergotamine-D-tartrate (3.28 mg) was dissolved in 50 mL ACN or MeOH to give a stock solution of 100 µM ergotamine. NH_4_OOCH (315.3 mg), (NH_4_)_2_CO_3_ (480.5 mg), and formic acid (188.7 µL) were placed in a 50 mL volumetric flask and filled up with ACN or MeOH to obtain a 100 µM stock solution. For the different experiments, 1 mL of the ergotamine-D-tartrate and the appropriate electrolyte/additive stock solutions were filled up to 10 mL with water and/or ACN/MeOH (Table 1). The reaction solution was pumped through the electrochemical cell at a velocity of 20 µL per minute, and the potential of the potentiate was ramped between 0 and 1200 mV at a scan rate of 5 mV/s. Direct measurements of the reaction products were carried out with the Agilent 6130 MS. For high-resolution mass spectra, samples were collected and measured via direct infusion at the Sciex TripleTOF 6600.

### 4.4. Synthesis of Norergotamine and Norergotaminine

Ergotamine-D-tartrate (10 mg, 15.2 µmol, 1.0 eq.) was suspended in 2 mL methanol and cooled down in an ice bath. After 15 min, mCPBA (3.75 mg, 16.7 µmol, 1.1 eq.) was added to the ice-cooled suspension, and the reaction was stirred for 1 h at room temperature or until no ergotamine was detected by LC-MS. During the reaction, the solid particles dissolved, creating a homogeneous solution. The magnetic stir bar was removed, and 1 molar hydrochloric acid in methanol (30.4 µL, 2.0 eq.), 5 g/L FeCl_3_∙6 H_2_O (41.4 µL, 0.05 eq.), and iron powder (8.4 mg, 150.2 µmol, 9.9 eq.) were added to the solution and shaken for 24 h at 35 °C. The color of the reaction changed from light yellow at the beginning to deep red at the end. After 24 h, 1 mL of solvent was removed under a constant flow of nitrogen. The solution was basified with a solution of 0.5 molar trisodium phosphate in water (76 µL, 2.5 eq.). The precipitate was removed by centrifugation, and the supernatant was purified by preparative LC (Table 4). The corresponding chromatogram is shown in Supplementary Material Figure S5.

The norergotamine/norergotaminine fractions were combined and dried overnight in a rotary vacuum concentrator at 35 °C and 11 mbar pressure. A total of 1.5 mg (2.6 µmol, 17.1%) of a mixture of norergotamine and norergotaminine was obtained.

*m*/*z* (measured) (M + H)^+^ = 568.2556 (theoretical (M + H)^+^: 568.2555, δ = 0.2 ppm)

### 4.5. Synthesis of Ergotamine-^13^CD_3_ and Ergotaminine-^13^CD_3_

A mixture of norergotamine and norergotaminine (1.5 mg, 2.6 µmol, 1.0 eq.) was dissolved in 300 µL acetone. *N*,*N*-Diisopropylethylamine (3.9 µmol, 1.5 eq.) and ^13^CD_3_-I (3.9 µmol, 1.5 eq.) were added to the solution and shaken at room temperature for 24 h. The solvent was removed in a rotary vacuum concentrator, and the residue was redissolved in 200 µL acetonitrile/methanol/water + 20 mM NH_3_ (40 v%/50 v%/10 v%). The crude mixture was purified via preparative LC (Table 5). The corresponding chromatogram is shown in Supplementary Material Figure S6.

*m*/*z* (measured) (M + H)^+^ = 586.2951 (theoretical (M + H)^+^: 586.2933, δ = 3.1 ppm)

The crude mixture was purified via preparative HPLC (Table 5) to yield a total of 1.33 mg (2.27 µmol; 14.9%) ergotamine-^13^CD_3_ and ergotaminine-^13^CD_3_.

## 5. Patents

The process described herein and the presented data for the preparation of *N*^6^-isotopically labeled ergot alkaloids are the subject of pending patent application EP24164604.1 and the German utility model with official reference number 202024101385.9.

## Figures and Tables

**Table 1:** Overview of the different working and auxiliary electrodes (GC: glassy carbon; BDD: boron-doped diamond; Pt: platinium; PEEK: polyether ether ketone) and concentrations of ammonium formate (NH_4_OOCH), formic acid (FA), and ammonium carbonate ((NH_4_)_2_CO_3_) used for the electrochemical reactions. The concentration of ergotamine was 10 µM in water/acetonitrile or water/methanol in different ratios (v%/v%) for each experiment.

Working/Auxiliary Electrode	Electrolyte	10 µM Ergotamine in Water/Acetonitrile or Water/Methanol (v%/v%)	N-Demethylated Product Detected
GC, BDD, and Pt/ PEEK	10 mM NH_4_OOCH	0/100, 20/80, and 50/50	Yes (only GC)
GC, BDD, and Pt/ PEEK	10 mM FA	0/100, 20/80, and 50/50	No
GC, BDD, and Pt/ PEEK	10 mM (NH_4_)_2_CO_3_	20/80 and 50/50	Yes (only GC)

**Table 2:** Parameters for the TripleTOF 6600 ESI-HR-MS/MS measurements.

Parameter—ESI Source		Parameter—Mass Spectrometer	
Temperature	300 °C	MS1	
Ion Source Gas 1	60 psi	Collision energy	5 V
Ion Source Gas 2	70 psi	Declustering Potential	80 V
Curtain Gas	25 psi	Mass range	400–800
Ionspray Voltage	5500 V	MS 2	
		Collision Energy	Rolling collision energy
		Collision Energy Spread	5 V
		Declustering Potential	80 V
		Mass range	100–800

**Table 3:** HPLC conditions: Phenomenex Kinetex Evo C18 (100 × 2.1 mm; 2.6 µm) column; flow rate: 0.6 mL/min; column oven temperature: 35 °C; injection volume: 5 µL; runtime: 15 min; eluents: H_2_O + 20 mM NH_3_; acetonitrile.

Time [min]	H_2_O + 20 mM NH_3_ [%]	Acetonitrile [%]
0	80	20
8.0	20	80
11.9	20	80
12	80	20
15	80	20

**Table 4:** Preparative LC conditions: Knauer Eurospher II 100-5 C18 P (250 × 4 mm; 5 µm) column; flow rate: 1 mL/mi’, column oven temperature: 35 °C; injection volume: 100 µL; runtime: 35 min; eluents: H_2_O + 20 mM NH_3_; acetonitrile; DAD wavelength: 254 nm.

Time [min]	H_2_O + 20 mM NH_3_ [%]	Acetonitrile [%]
0	66	34
20	66	34
20.1	0	100
25	0	100
25.1	66	34
31	66	34

**Table 5:** Preparative LC conditions: Knauer Eurospher II 100-5 C18 P (250 × 4 mm; 5 µm) column; flow rate: 1 mL/min; column oven temperature: 35 °C; injection volume: 100 µL; runtime: 35 min; eluents: H_2_O + 20 mM NH_3_; acetonitrile; DAD wavelength: 254 nm.

Time [min]	H_2_O + 20 mM NH_3_ [%]	Acetonitrile [%]
0	62	38
14	62	38
16	35	65
22	35	65
22.1	0	100
27	0	100
27.1	62	38
33	62	38

## Data Availability

Data are contained within the article and Supplementary Materials.

## Supplementary Materials

The following supporting information can be downloaded at https://www.mdpi.com/article/10.3390/toxins16040199/s1: Table S1: Formula, theoretical mass (*m*/*z*), observed mass, and deviation [ppm] for the main fragment ions of ergotamine-^13^CD_3_; Figure S1: Potential fragmentation of ergotamine/-inine-^13^CD_3_; Figure S2: Full HR-MS/MS spectra of unlabeled ergotamine and labeled ergotamine-^13^CD_3_; Figure S3: Full HR-MS/MS spectra of unlabeled ergotaminine and labeled ergotaminine-^13^CD_3_; Figure S4: Full extracted-ion chromatogram of unlabeled ergotamine/-inine and isotopically labeled ergotamine/-inine-^13^CD_3_; Figure S5: Preparative HPLC-DAD chromatogram for the purification of norergotamine and norergotaminine; Figure S6: Preparative HPLC-DAD chromatogram for the purification of ergotamine-^13^CD_3_ and ergotaminine-^13^CD_3_.
