# Supplementary material for: First Synthesis of Ergotamine-13CD3 and Ergotaminine-13CD3 from Unlabeled Ergotamine
Source: Toxins (Basel). 2024 Apr 20;16(4):199. doi: 10.3390/toxins16040199 (PMC11053779; doi:10.3390/toxins16040199)
Supplement: Supplementary file 1 [file toxins-16-00199-s001.zip › toxins-2959457-supplementary.pdf]

Supplementary Materials: First Synthesis of Ergotamine-$^{13}CD_3$ and Ergotaminine-$^{13}CD_3$ from Unlabeled Ergotamine

Sven-Oliver Herter, Hajo Haase and Matthias Koch

Table S1: Formula, theoretical mass (*m*/*z*), observed mass and deviation Δm/m [ppm] for the main fragment ions of ergotamine-$^{13}CD_3$.

Formula	Theoretical mass [M+H]+	Measured mass [M+H]+	Δm/m [ppm]
$^{13}CC_{32}H_{33}D_3N_5O_5^+$	586.2933	586.2933	0 - recalibrated
$^{13}CC_{32}H_{31}D_3N_5O_4^+$	568.2827	568.2817	-1.8
$^{13}CC_{31}H_{31}D_3N_5O_5^+$	540.2878	540.2863	-2.6
$^{13}CC_{18}H_{15}D_3N_3O_2^+$	324.1615	324.1608	-2.3
$C_{17}H_{20}N_3O_3^+$	314.1499	314.1490	-2.9
$C_{17}H_{17}N_2O_3^+$	297.1234	297.1231	-1.0
$^{13}CC_{17}H_{15}D_3N_3O^+$	290.1666	290.1660	-2.0
$C_{17}H_{13}N_2O_2^+$	277.0972	277.0970	-0.6
$^{13}CC_{15}H_{15}D_3N_3O^+$	272.1666	272.1664	-0.8
$^{13}CC_{15}H_{12}D_3N_2O^+$	255.1401	255.1394	-2.6
$^{13}CC_{14}H_{12}D_3N_2^+$	227.1452	227.1449	-1.1
$C_{14}H_{10}NO^+$	208.0757	208.0752	-3.3

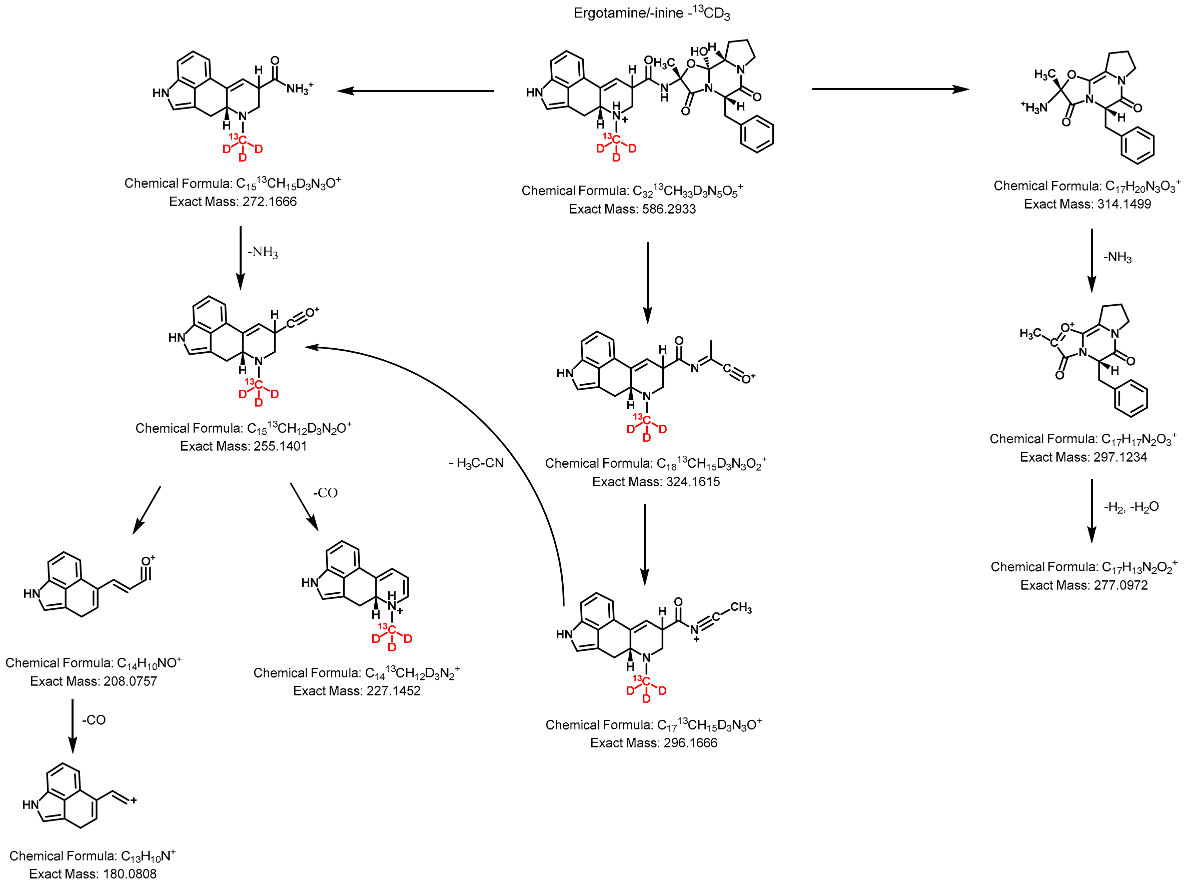

Figure S1. Potential structures of ergotamine/-inine-$^{13}CD_3$ product ions produced by MS/MS. A structural proposal for the compound with the theoretical mass m/z 277.0972 could not be made due to complex fragmentation. Therefore, only the calculated chemical formula is provided.

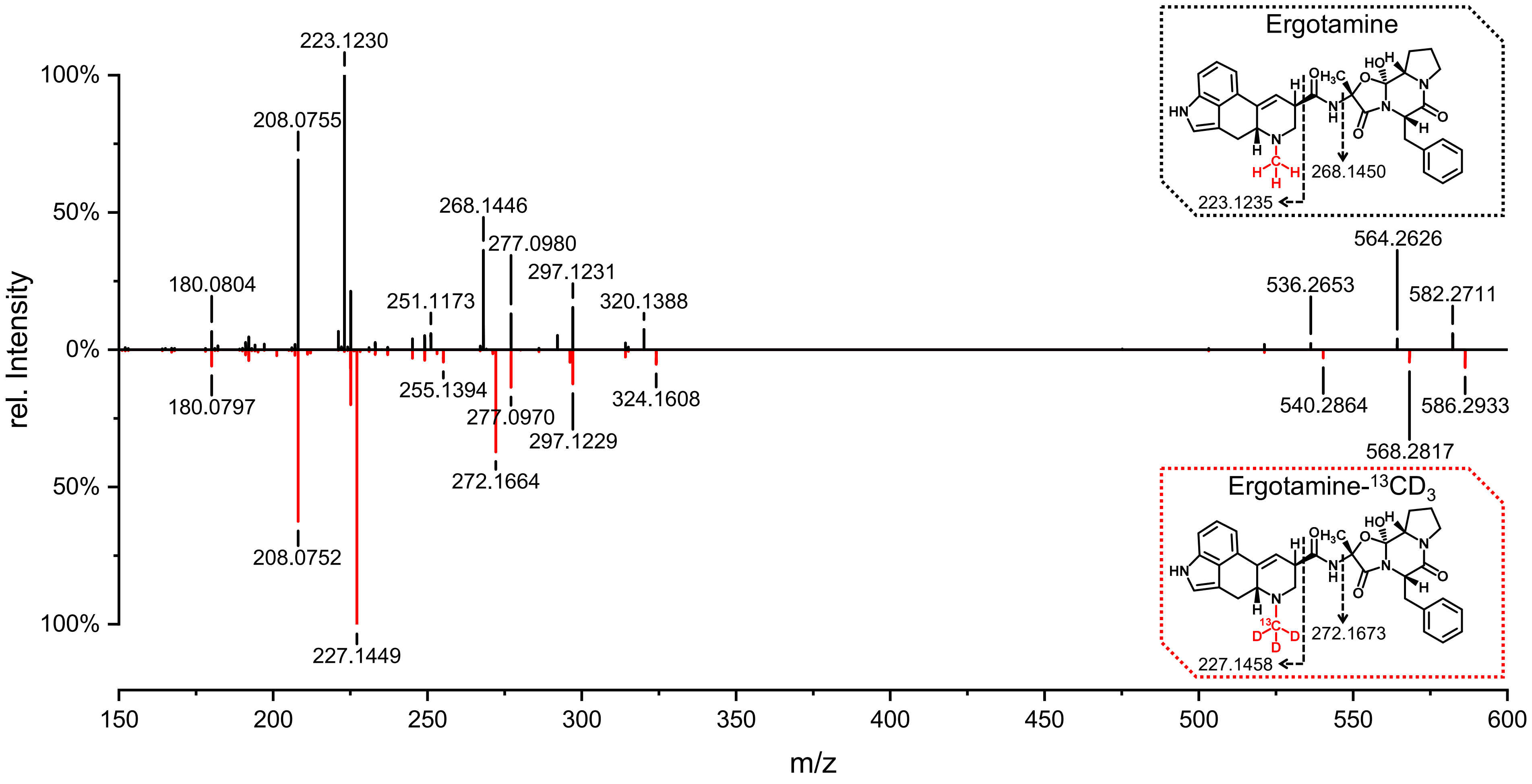

Figure S2. High resolution tandem mass spectra of unlabeled ergotamine (black) and isotopically labelled ergotamine-$^{13}CD_3$ (red).

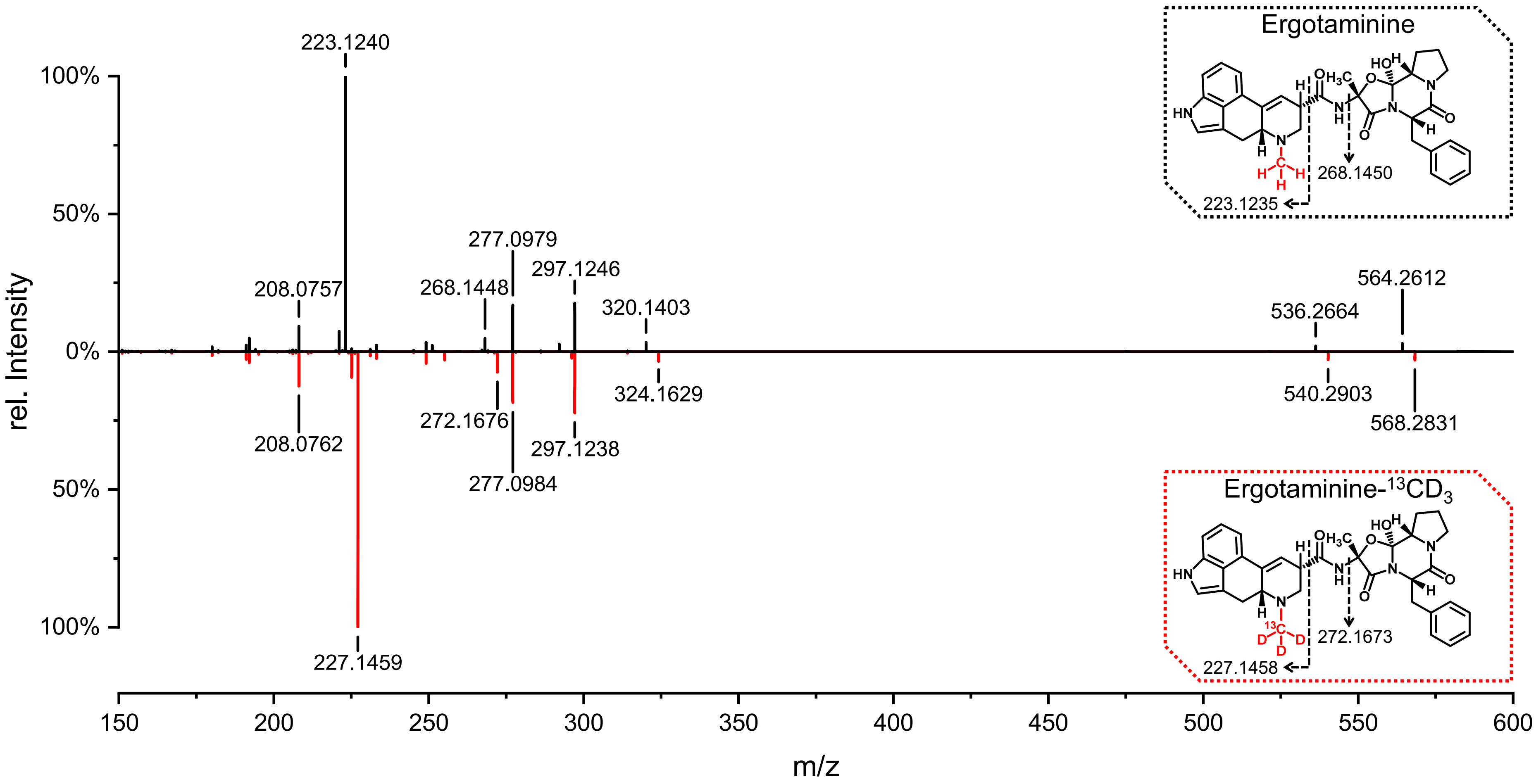

Figure S3. High resolution tandem mass spectra of unlabeled ergotaminine (black) and isotopically labelled ergotaminine-$^{13}CD_3$ (red).

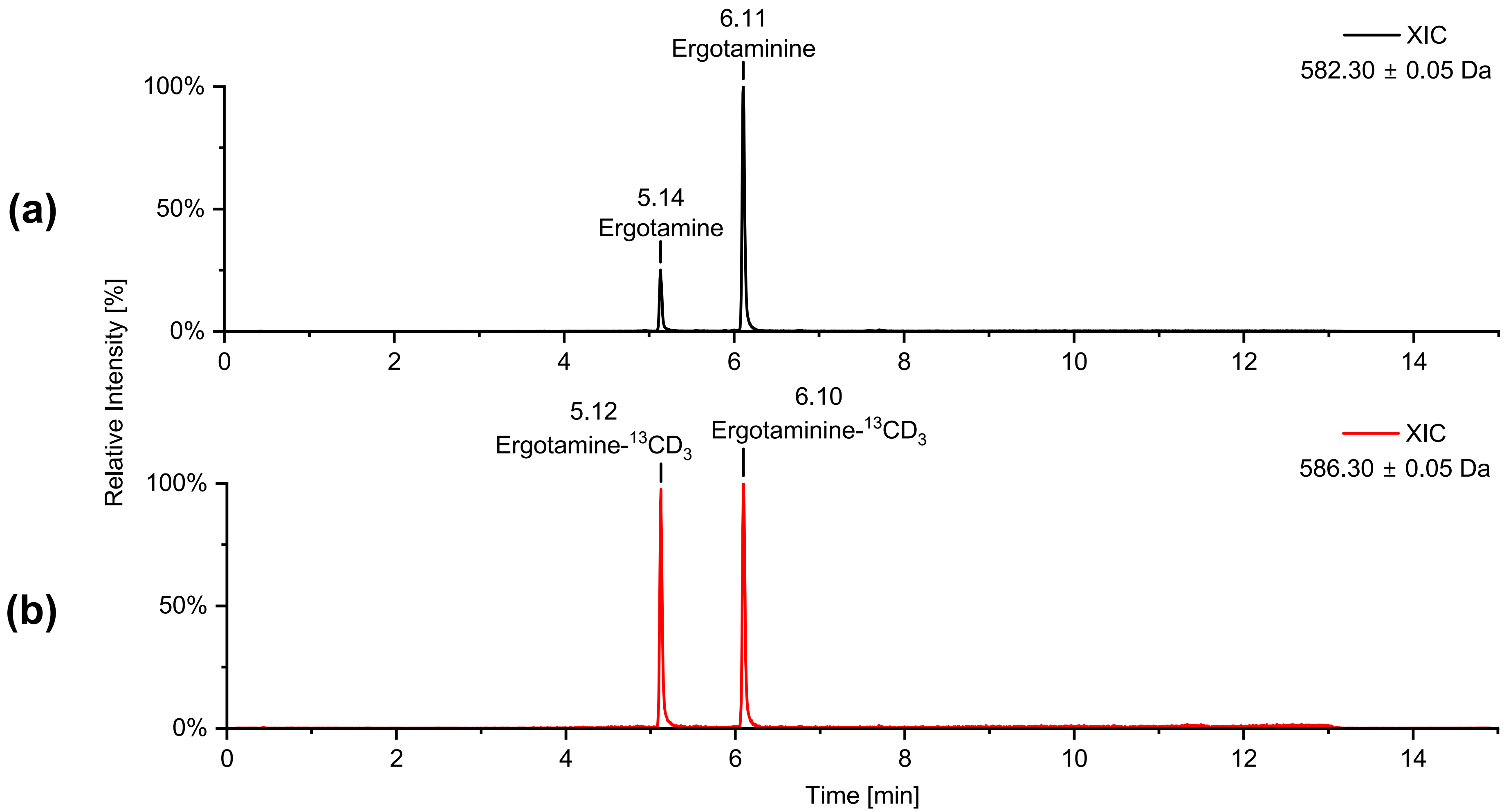

Figure S4. Extracted ion chromatogram (XIC) of **a)** unlabeled ergotamine and ergotaminine **b)** isotopically labelled ergotamine-$^{13}CD_3$ and ergotaminine-$^{13}CD_3$.

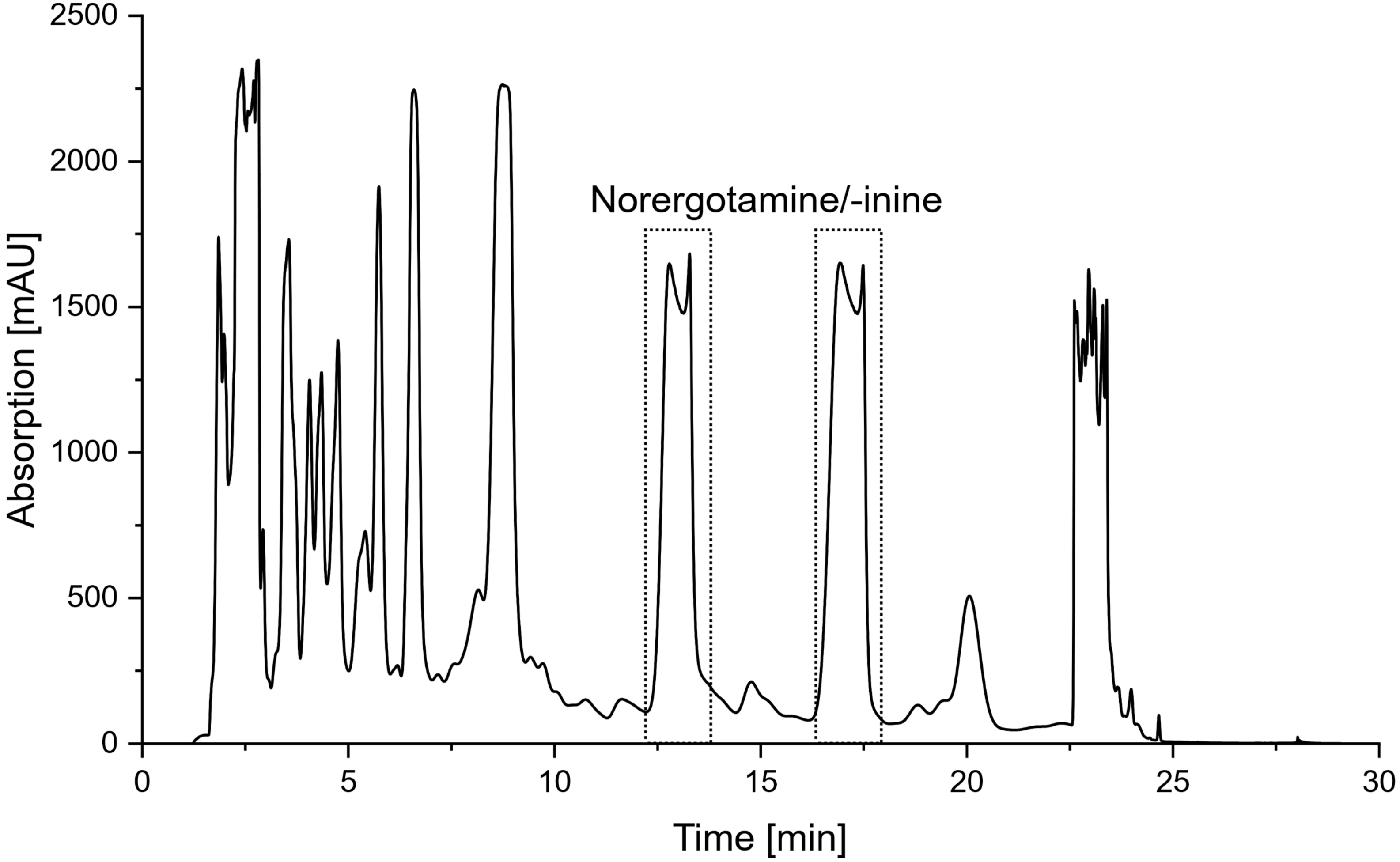

Figure S5. Preparative HPLC-DAD chromatogram for the purification of norergotamine and norergotaminine.

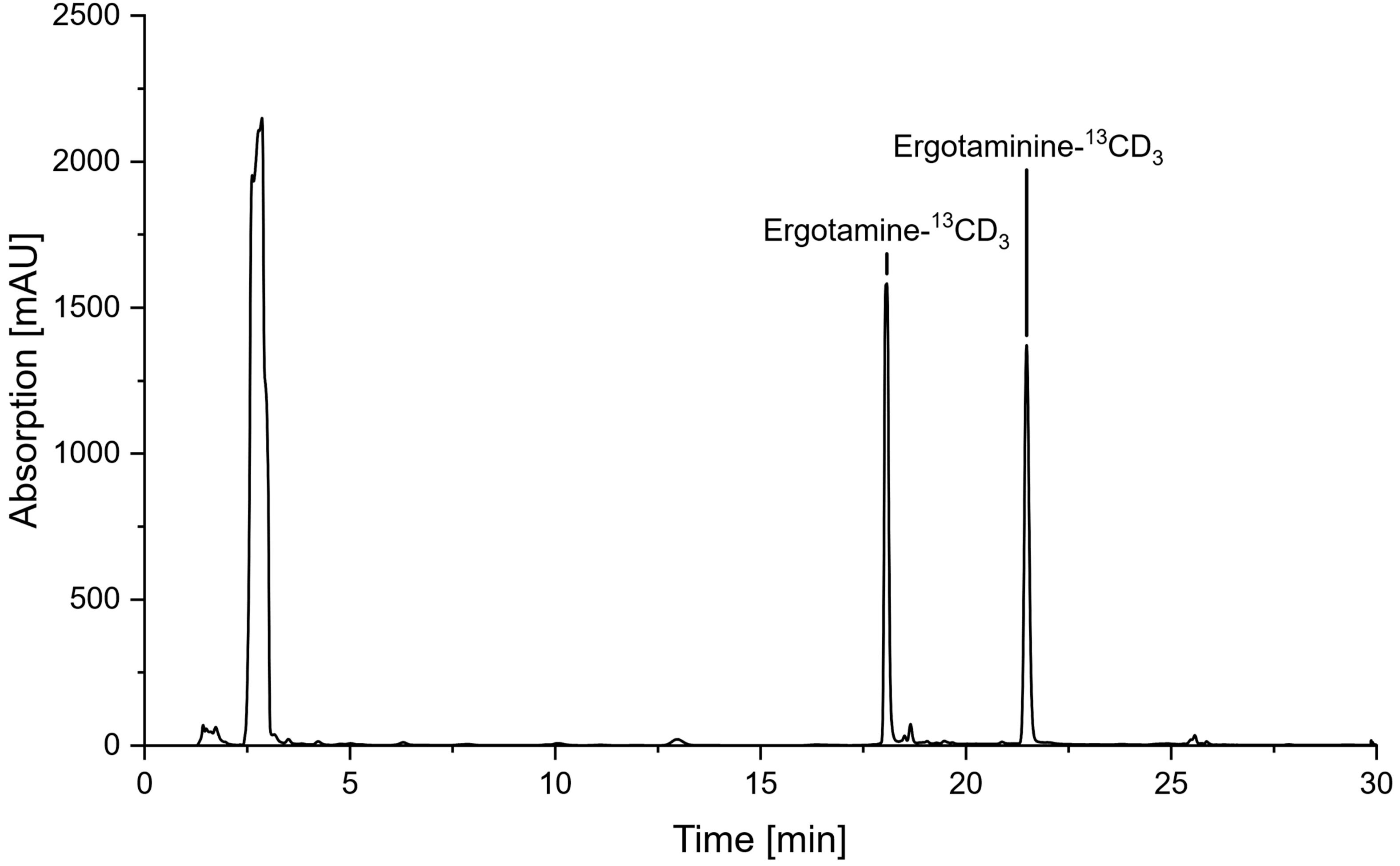

Figure S6. Preparative HPLC-DAD chromatogram for the purification of ergotamine-$^{13}CD_3$ and ergotaminine-$^{13}CD_3$.
